# Augmented Reality for Smoking Cessation: Development and Usability Study

**DOI:** 10.2196/21643

**Published:** 2020-12-31

**Authors:** Christine Vinci, Karen O Brandon, Marloes Kleinjan, Laura M Hernandez, Leslie E Sawyer, Jody Haneke, Steven K Sutton, Thomas H Brandon

**Affiliations:** 1 Department of Health Outcomes and Behavior Moffitt Cancer Center Tampa, FL United States; 2 Department of Oncologic Sciences University of South Florida Tampa, FL United States; 3 Department of Psychology University of South Florida Tampa, FL United States; 4 Trimbos Institute Utrecht Netherlands; 5 Department of Interdisciplinary Social Science Utrecht University Utrecht Netherlands; 6 Haneke Design Tampa, FL United States; 7 Department of Biostatistics and Bioinformatics Moffitt Cancer Center Tampa, FL United States

**Keywords:** augmented reality, smoking cessation, cue exposure therapy, cue reactivity, behavior change, smoking, smartphone app, mobile phone

## Abstract

**Background:**

The recent widespread availability of augmented reality via smartphone offers an opportunity to translate cue exposure therapy for smoking cessation from the laboratory to the real world. Despite significant reductions in the smoking rates in the last decade, approximately 13.7% of the adults in the United States continue to smoke. Smoking-related cue exposure has demonstrated promise as an adjuvant therapy in the laboratory, but practical limitations have prevented its success in the real world. Augmented reality technology presents an innovative approach to overcome these limitations.

**Objective:**

The aim of this study was to develop a smartphone app that presents smoking-related augmented reality images for cue exposure. Smokers provided feedback on the images and reported on the perceived urge to smoke, qualities of reality/coexistence, and general feedback about quality and functioning. The feedback was used to refine the augmented reality images within the app.

**Methods:**

In collaboration with an augmented reality design company, we developed 6 smoking-related images (cigarette, lighter, ashtray, lit cigarette in ashtray, etc) and 6 neutral images similar in size or complexity for comparison (pen, eraser, notebook, soda bottle with droplets, etc). Ten smokers completed a survey of demographic characteristics, smoking history and behavior, dependence on nicotine, motivation to quit smoking, and familiarity with augmented reality technology. Then, participants viewed each augmented reality image and provided ratings on 10-point Likert scales for urge to smoke and reality/coexistence of the image into the scene. Participants were also queried with open-ended questions regarding the features of the images.

**Results:**

Of the 10 participants, 5 (50%) had experienced augmented reality prior to the laboratory visit, but only 4 of those 5 participants used augmented reality at least weekly. Although the sample was small (N=10), smokers reported significantly higher urge to smoke after viewing the smoking-related augmented reality images (median 4.58, SD 3.49) versus the neutral images (median 1.42, SD 3.01) (Z=–2.14, *P*=.03; *d*=0.70). The average reality and coexistence ratings of the images did not differ between smoking-related and neutral images (all *P*>.29). Augmented reality images were found on average to be realistic (mean [SD] score 6.49 [3.11]) and have good environmental coexistence (mean [SD] score 6.93 [3.04]) and user coexistence (mean [SD] score 6.38 [3.27]) on the 10-point scale. Participant interviews revealed some areas of excellence (eg, details of the lit cigarette) and areas for improvement (eg, stability of images, lighting).

**Conclusions:**

All images were generally perceived as being realistic and well-integrated into the environment. However, the smoking augmented reality images produced higher urge to smoke than the neutral augmented reality images. In total, our findings support the potential utility of augmented reality for cue exposure therapy. Future directions and next steps are discussed.

## Introduction

Augmented reality is an emerging technology that could provide a novel and exciting treatment strategy for substance use disorders [1]. Augmented reality superimposes virtual, digital objects into the real world environment, often via a smartphone or a tablet [2]. The advantages of augmented reality as a treatment modality include its scalability and application in the real world environment (vs treatment in a clinic). To date, augmented reality has been used primarily in gaming and retail phone apps as well as for training purposes in the medical field [3,4]. With respect to behavioral treatments, augmented reality has mainly been tested for the treatment of specific phobias via cue exposure, with promising outcomes [5]. We propose that augmented reality might be particularly well suited for the treatment of substance use disorders, and we begin by focusing on tobacco dependence.

Cigarette smoking is the leading preventable cause of morbidity and mortality in the United States [6]. Although tobacco use has declined over the past 50 years, smoking prevalence was reported to be 13.7% among adults in the United States in 2018 [7]. As such, developing more effective cessation interventions remains a public health priority.

There is a specific need for cessation interventions that reduce barriers to dissemination and implementation, and mobile health holds particular promise in this regard [8-11]. Moreover, harnessing recent advances in technology may increase the uptake of cessation treatment, while also targeting identified roadblocks to cessation. For example, in 2019, 81% of Americans reported owning a smartphone, which was up from 35% from only 7 years earlier, with 70% ownership even among low-income populations [12]. Further, among all smartphone owners in 2018, almost half reported using an app to monitor their health [13]. Thus, novel interventions that leverage advances in technology may be an ideal way to increase the reach and efficacy of smoking cessation treatments.

Through the display of virtual smoking cues superimposed upon smokers’ natural environment, augmented reality appears ideal for improving the long-term effects of cue exposure. Cue exposure therapy is based on the principles of Pavlovian conditioning [14,15], which when applied to drug behavior consists of the repeated presentation of smoking-related cues (ie, conditioned stimuli) without access to nicotine (ie, the unconditioned stimulus), resulting in the extinction of craving (ie, the conditioned response). Thus, cue exposure therapy typically consists of presenting a drug cue (eg, cigarette) to individuals multiple times while not allowing them to engage in the typical drug behavior (in this case, smoking), with the goal of extinguishing the urge to smoke in response to these cues. The ultimate clinical goal is to reduce the risk of smoking relapse when individuals encounter similar cues in their environment [16,17]. Cue exposure treatments have demonstrated efficacy in decreasing craving in the laboratory, but these effects are often short-lived as they do not generalize well beyond the extinction setting (eg, laboratory or clinic) into the real world [18,19]. Augmented reality allows the entire cue exposure session to take place in the real world, which could greatly enhance the efficacy of cue exposure treatment. Additionally, the COVID-19 pandemic has elevated the utility of mobile health interventions that move treatment from a clinic to a patient’s own environment.

To our knowledge, there have been no previous studies examining the potential of augmented reality for cue exposure treatment for substance use disorders. Thus, this paper presents the initial phase of designing and testing the usability of augmented reality stimuli in a small sample of smokers. The primary aim was to receive feedback on the stimuli and modify as needed.

## Methods

### Study Participants

Ten participants were recruited in October and November of 2019. Inclusion criteria were as follows: (1) ≥18 years of age; (2) currently smoking ≥3 cigarettes per day during the past year; (3) having a breath carbon monoxide (CO) level ≥5 ppm to verify smoking status; (4) motivated to quit smoking; (5) having a valid home address in the local area; (6) having a functioning telephone number; and (7) the ability to speak, read, and write in English. Exclusion criteria were regular use (eg, on more than one-third of tobacco use occasions) of other tobacco products or a household member already enrolled in the study.

### Procedures

#### Smartphone App and Stimuli Development

A smartphone app was developed as a platform for presenting cue-reactivity stimuli as augmented reality images. The stimuli were consistent with the existing cue-reactivity literature and commonly used cues within the field [20-24]. Proximal smoking cues (eg, cigarettes, lighters, ashtrays) were developed, and for comparison purposes, we selected neutral cues that were similar in size and complexity to the smoking cues whenever feasible (eg, pencil, eraser, notebook). Single and compound cues, in conjunction with one motion cue, were used for smoking-related and neutral images. Neutral items represented the general category of common school or office supplies, apart from a moving image (a soda bottle with condensation). Table 1 lists the type of cue (single, compound, motion item) for each category of cue (smoking-related or neutral) that was used.

**Figure 1:**
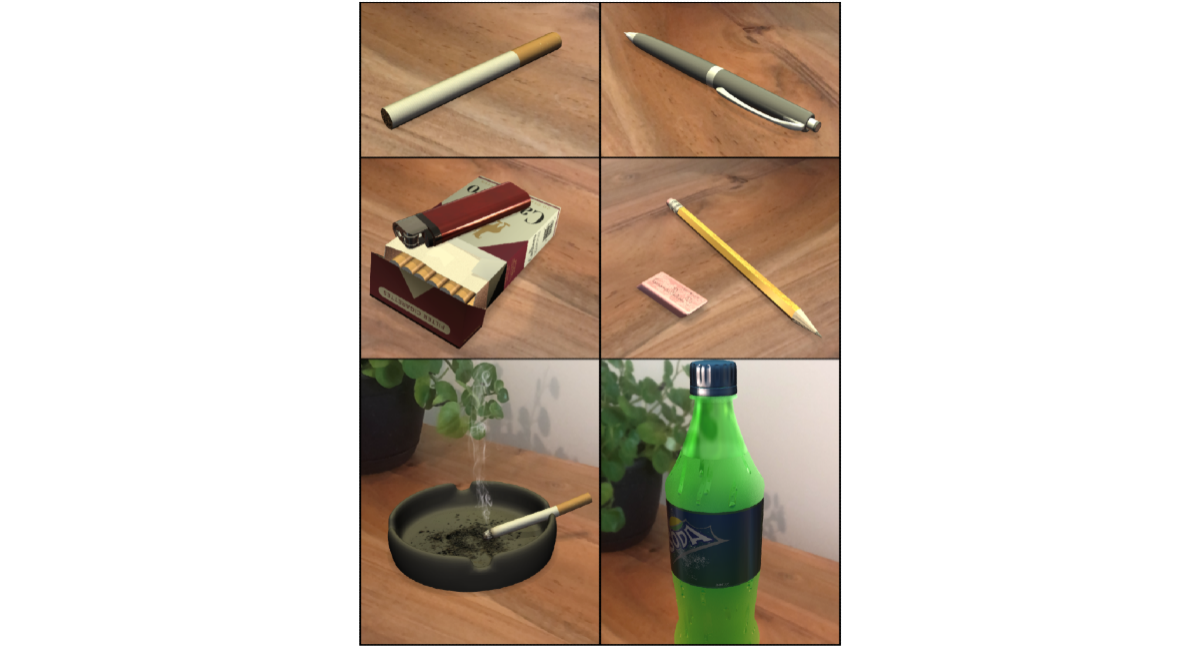
A subset of digitally created augmented reality smoking-related and neutral cues.

**Figure 2:**
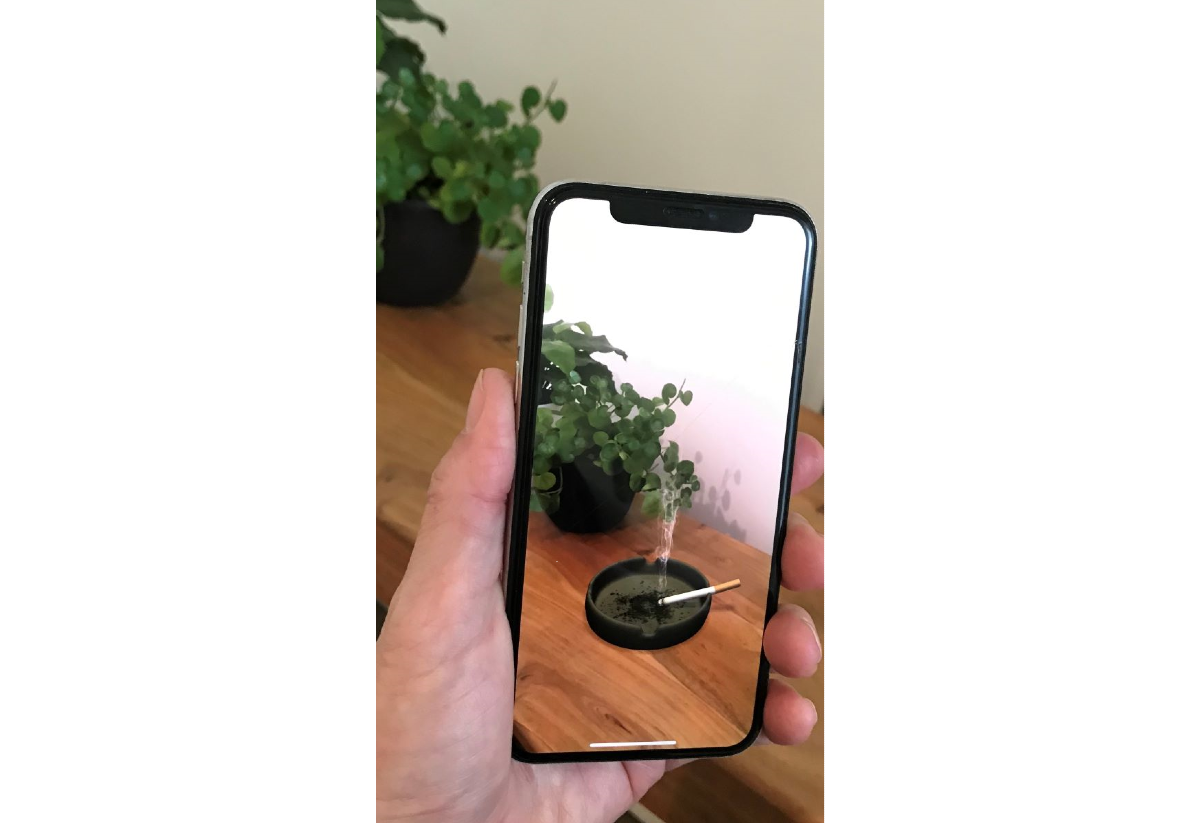
Digitally created augmented reality smoking image superimposed on a user’s table in real time. This image includes the motion feature of smoke rising from the cigarette tip.

The augmented reality design company, Haneke Design, created 12 initial images by using the above guidelines (Table 1). Some images (assets) were provided by the Unity [25] platform software, while others were purchased (ie, animated cigarette smoke). Image rendering was conducted to create images not available in the Unity library, including a pack of cigarettes and a soda bottle. In such instances, rendering was done using extrusion modeling and an additional app, Blender v.2.80 [26], which is a free and open-source 3D computer graphics software toolset for visual effects, motion graphics, and interactive 3D apps. The developer also used photos of a plate with a cigarette to render an ashtray since the asset was not available in the Unity library. Other assets, including the soda bottle and cigarette pack, were also captured using digital photography and by using extrusion modeling and the Blender software app to provide realistic augmented reality images. Initial steps by the research team included contacting the developers to discuss features, images, and overall design of the app. After all the features and images were determined, the company developed and deployed the initial app to the study team for review and staff testing. The research team tested the images and provided feedback regarding item features (color, orientation, size, texture, etc) through an iterative process until the images were deemed satisfactory. Next, the developers incorporated the images in a smartphone compatible app created to deliver the images to the participants. For example, features such as participant identification number, timed presentation of the augmented reality images, user-friendly urge and reality/coexistence ratings, and data (ID, order of viewing, ratings) storage and export capabilities were included. Figure 1 and Figure 2 are pictures of several of the final images. The video of an image placement and rating can be found in Multimedia Appendix 1.

**Table 1:** Types of cues for smoking and neutral augmented reality images.

Type of cue, subtype		Smoking images	Neutral images
**Single cues**			
	Single cue 1	Cigarette	Pen
	Single cue 2	Pack of cigarettes	Notepad
**Compound cues**			
	Compound cue 1	Pack of cigarettes with ashtray	Pencil and eraser
	Compound cue 2	Cigarette and lighter	Pencil with notepad
	Compound cue 3	Pack of cigarettes with lighter	Sticky notes and pen
Motion cue		Ashtray with lit cigarette emitting smoke	Soda bottle with condensation droplets and effervescence

#### Participant Recruitment and In-Person Session

Participants were recruited through referrals from previous studies within our laboratory. Individuals were contacted by phone and they completed a brief phone screen for eligibility assessment. Eligible participants were invited to attend an in-person session, where the informed consent process was completed. After participants provided consent, a breath CO sample was collected to confirm smoking status. Participants completed a brief survey of demographics, smoking history, self-reported nicotine dependence, motivation to quit smoking, and familiarity with augmented reality. Participants were instructed on app use through an experimenter-guided user manual. The manual detailed how to position the images on the table and showed examples of sequential screenshots. Participants were provided with an Apple 10XR iPhone with the augmented reality app to view each of the 12 images. Each participant was assigned a random order for viewing the images.

**Figure 3:**
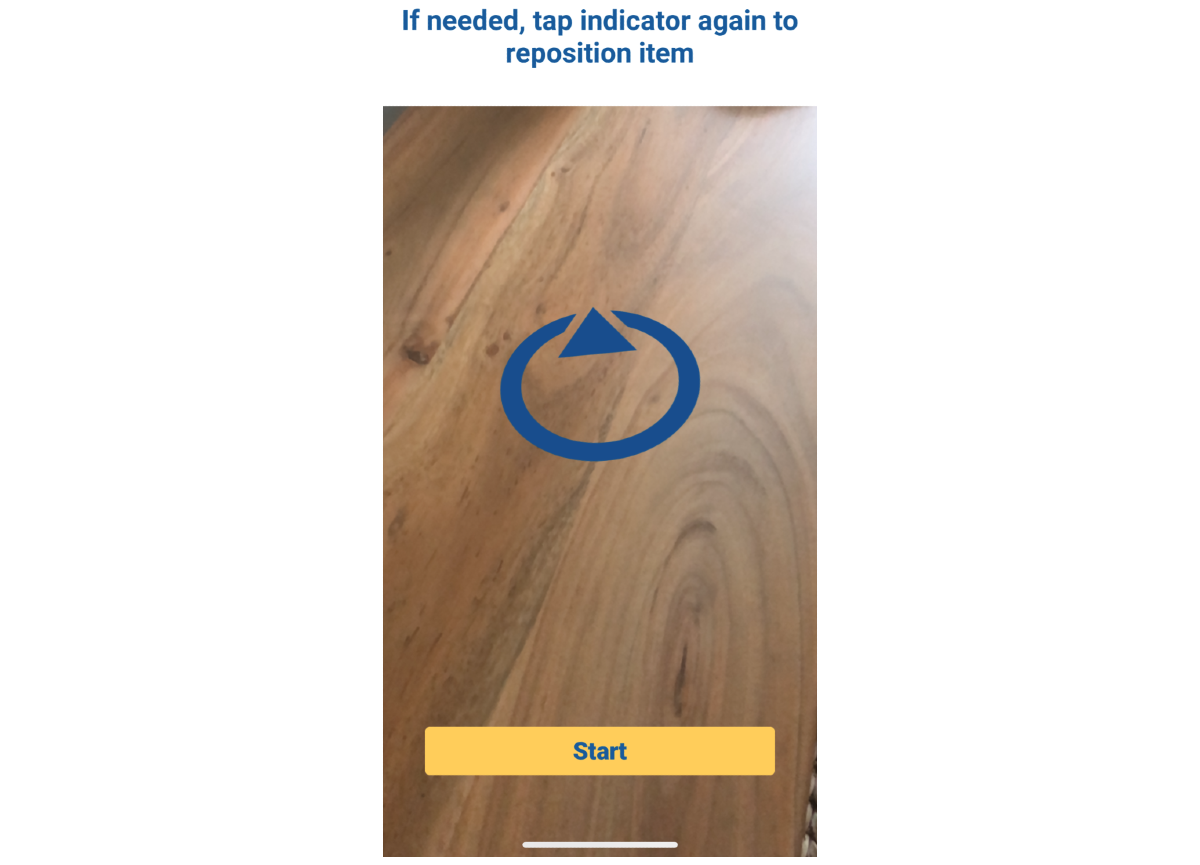
Screenshot of the digitally created blue placement circle.

**Figure 4:**
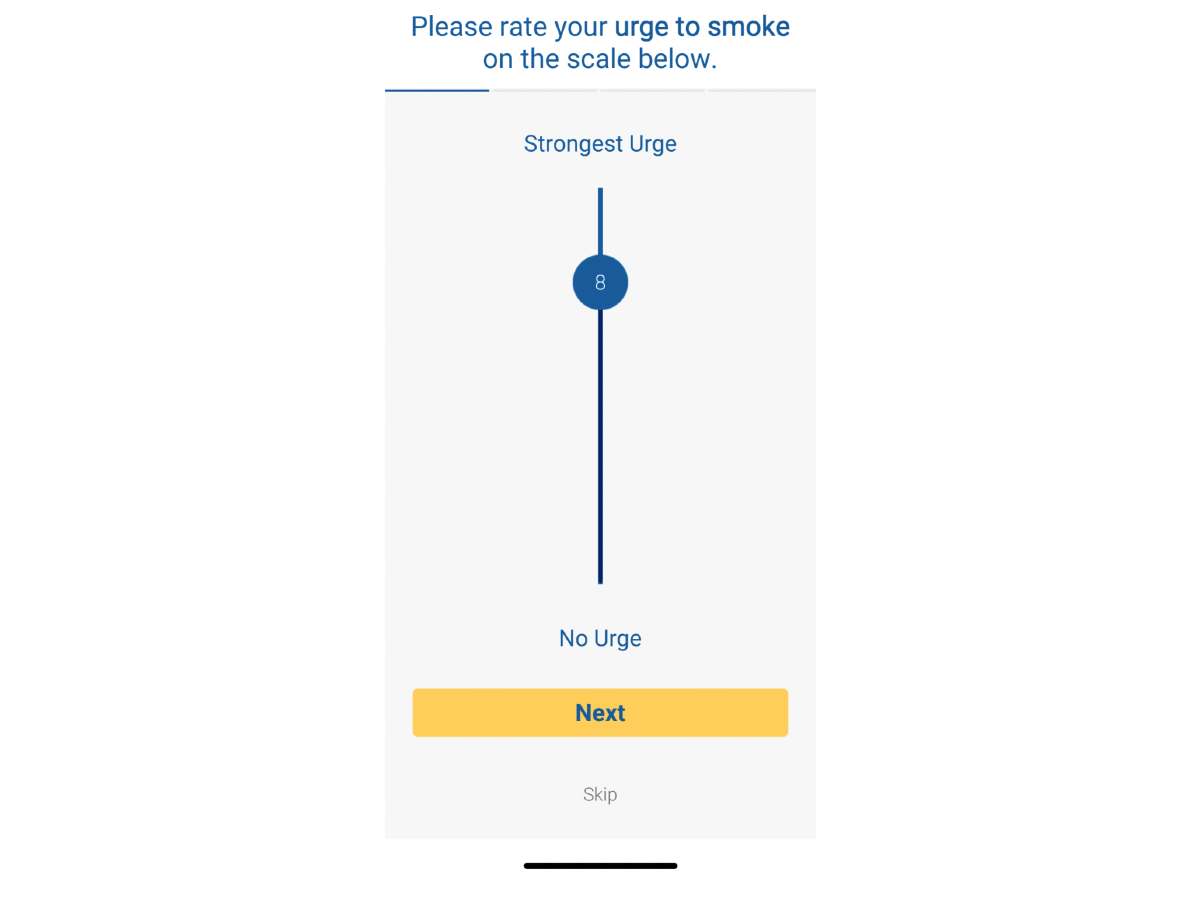
Screenshot of a smoking urge rating after viewing the augmented reality image.

After the experimenter selected the first image to be viewed, the app guided the participant on image placement upon a table that was empty apart from a tissue box placed to provide context for the augmented reality image. Participants were instructed to place an augmented reality–generated blue arrow (Figure 3) anywhere on the table. Once the blue arrow was placed where they liked, participants tapped on the blue arrow, which allowed the selected image to appear in the center of the arrow. Once the participants were satisfied with the placement, the image was locked into place by pressing the “Start” button that appeared at the bottom of the screen. The augmented reality image was visible for 1 minute, during which the participant was free to view the augmented reality image from multiple directions and distances. After 1 minute, the image disappeared from the screen and the app displayed Likert scale questions to obtain ratings on smoking urge and image reality/coexistence (Figure 4). Next, the experimenter asked open-ended questions to obtain general feedback on the image (discussed in further detail below). Once completed, the next augmented reality image was selected for viewing, and the process was repeated for each of the 12 augmented reality images. Sessions lasted approximately 1 hour, and participants were compensated US $35 for their time. All procedures were approved by the Advarra Institutional Review Board.

### Measures

#### Baseline Variables

Self-report items assessed demographic variables (age, race, ethnicity, sex, and education with 8 categories from no high school to professional degree, and household income). Smoking and other tobacco use in the past month was also assessed (eg, “In the past month, how often have you smoked cigarettes on average?” with response categories of “no use in the past month,” “1-5 times per month,” “1-3 days a week,” “4-6 days a week,” and “7 days a week”). The number of previous cessation attempts (“In the past month, how many cigarettes per day have you smoked on average?”), motivation to quit smoking, and nicotine dependence were also assessed. Motivation to quit was assessed by 2 Yes/No items asking about plans to quit smoking in the next 3 and 6 months, 1 Likert scale item assessing confidence that they could stay off cigarettes for good if they tried to quit (1=“Not at all” to 5=“Very”), 1 Likert item rating how strongly they agree with the statement, “I am committed to quitting smoking” (1=“Not at all” to 5=“Very”), and the Contemplation Ladder, [27] a 10-point scale (1=“No thoughts of quitting smoking cigarettes” to 10=“Taking action to quit smoking cigarettes”). Tobacco dependence was measured by the 6-item Fagerström Test for Nicotine Dependence scale (FTND) [28]. The FTND items assess the number of cigarettes smoked per day, time to first smoked cigarette after waking, difficulty not smoking in places where smoking is prohibited, most difficult cigarette to give up (first one in the morning or any other), smoking more within the first 2 hours of the day, and smoking even when ill. We also assessed participants’ familiarity and past use of augmented reality by asking “Have you ever used any kind of augmented reality (AR) app before? (eg, augmented reality feature in Pokémon Go, Ikea or other furniture app, Snapchat filters, Google Sky Map, etc)"; the answer options were Yes/No/Don’t know and by asking “How frequently do you use augmented reality (AR) apps?” (ie, answer options were 7 days a week/4-6 days a week/1-3 days a week/1-5 times a month/less than once a month/Never/Don’t know).

#### Ratings of Augmented Reality Images

Urge (craving) to smoke was rated on a Likert scale (1=absolutely no urge to smoke to 10=strongest urge to smoke) (Figure 4). Three items assessed the quality of the augmented reality experience [29] using 10-point Likert scales: (1) a reality item captured the perceived realism of the augmented reality object, “How real did the object seem to you?” from 1=Not all to 10=Very Real; (2) an environment coexistence item assessed the integration of the object into the surrounding environment, as seen on the smartphone screen, “How well did the object appear to be part of the scene?” from 1=Not at all to 10=Very Well; and (3) user coexistence captured the degree to which the user felt in the presence of the object, “How much did you feel the object was right there in front of you?” from 1=Not at all to 10=Very Much.

#### Additional Feedback on Images

After viewing each image, participants were asked for their open-ended feedback with the following questions: “What were your initial thoughts about the item?”, “Is there anything else that seems wrong, off, or not quite right about the item?”, “Do you have any other feedback that you think would improve the item?” Additional specific questions were asked about the 2 images with motion aspects (ie, lit cigarette in ashtray and soda bottle with droplets). Finally, we asked participants if they could recommend additional images that might elicit urges to smoke.

### Statistical Analysis

Although this development study was not powered for inferential statistics, we nevertheless compared the median urge scores of smoking versus neutral augmented reality images by using the Wilcoxon signed-rank test after calculating each participant’s mean rating across smoking and neutral images. Cue reactivity would be indicated by higher urges elicited by the smoking images compared to the neutral images. Evidence of such cue reactivity is a necessary condition for subsequent cue exposure therapy designed to reduce conditioned urge. We also compared smoking and neutral images with respect to realism/coexistence.

## Results

### Sample Characteristics

Thirty-nine people were screened for eligibility. Seventeen individuals met the eligibility criteria, 15 were scheduled for a session, and 11 consented. One participant who gave consent did not meet the CO eligibility and thus did not participate in the session; therefore, only 10 participants were included in the analyses. The descriptive characteristics of the participants are summarized in Table 2. Of the 10 participants, 6 (60%) were males and the majority was white, non-Hispanic; 8 (80%) of them had an annual household income of less than US $40,000. All participants were daily smokers and smoked an average of 18 cigarettes per day. Participants’ nicotine dependence as indexed by the FTND demonstrated a full range of dependence levels in our sample. The average participant motivation to quit was moderately high, with all but 1 participant planning a quit attempt in the next 6 months.

**Table 2:** Statistical summary of the descriptive characteristics of the participants (N=10).

Descriptive characteristics, variable, subvariable			Values
**Demographics**			
	Age (years), mean (SD)		50.70 (9.84)
	Male, n (%)		6 (60)
	Latino, n (%)		3 (30)
	Income <US $40,000, n (%)		8 (80)
	Education < High school, n (%)		3 (30)
	**Race, n (%)**		
		White	8 (80)
		Black	2 (20)
**Smoking characteristics**			
	Cigarettes per day, mean (SD)		18.30 (6.57)
	FTND^a^ total, mean (SD)		4.80 (2.39)
	**Nicotine dependence, n (%)**		
		Low dependence	1 (10)
		Low-to-moderate dependence	4 (40)
		Moderate dependence	3 (30)
		High dependence	2 (20)
	Other household members smoke, n (%)		3 (30)
	**Plans to quit smoking, n (%)**		
		Within 6 months	9 (90)
		Within 3 months	7 (70)
	**Quitting motivation, mean (SD) scores**		
		Confidence in quitting (scale 1-5)	2.90 (1.37)
		Commitment to quitting smoking (scale 1-5)	3.70 (1.06)
		Contemplation ladder (scale 1-10)	6.10 (2.38)
**Augmented reality ever used, n (%)**			5 (50)
	**Frequency of use of those who had used augmented reality, n (%)**		
		4-6 days/week	1 (20)
		1-5 days/week	1 (20)
		Less than once/month	3 (60)

^a^FTND: Fagerström Test for Nicotine Dependence.

### Ratings of Augmented Reality Images

**Figure 5:**
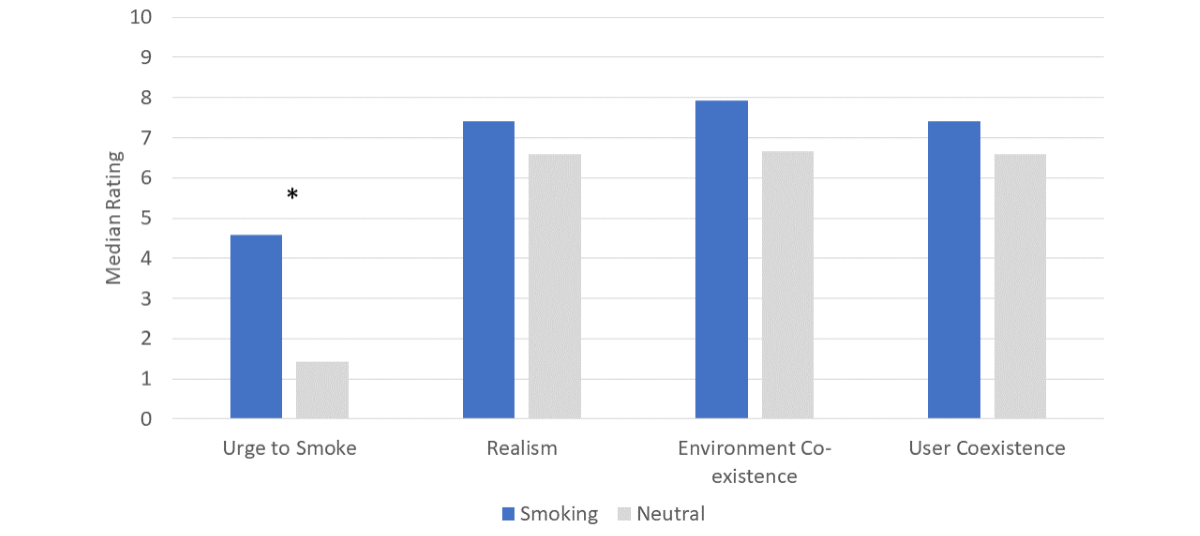
Median ratings of urge to smoke and reality/coexistence measures across image categories. *P=.03.

Figure 5 shows the median ratings of urge to smoke and reality/coexistence across the 6 smoking-related images when compared to the median ratings across the 6 neutral images. As expected, participants reported higher ratings of smoking urge in response to the smoking stimuli (median 4.58) compared to neutral stimuli (median 1.42) (Z=–2.14, *P*=.03), with a moderate effect size (Cohen *d*=0.70). No significant differences were found in the average ratings of reality/coexistence between the categories of augmented reality images (*P*>.29). The average ratings of augmented reality images indicated that these images were realistic (mean [SD] score 6.49 [3.11]) and have both good environmental coexistence (mean [SD] score 6.93 [3.04]) and user coexistence (mean [SD] score 6.38 [3.27]). Multimedia Appendix 2 and Multimedia Appendix 3 show the mean ratings of each individual smoking-related and neutral cue (respectively) urge to smoke and reality/coexistence.

### Participant Usability Feedback

Across the 12 items and 10 participants (120 reports), there were 18 mentions of the image moving, rolling, or floating above or off the table, 16 reports of the image items being either too big or too small, 16 comments regarding shadow/lighting oddities, 20 mentions of color inconsistencies or dislikes, 8 reports of pixilation problems, 16 mentions that the image or details of the image were not the same as their brand (smoking-related images only), and 21 comments regarding details that were missing (eg, no. 2 on the pencil, ashtray too dirty or too clean, lack of brand name on the cigarette filter). Although we were not focused on capturing the positive aspects of the stimuli per se, many participants offered comments referencing item realism (eg, “seemed realistic”), level of detail (eg, “pretty good graphics,” “really good, seemed detailed”), and urge response (eg, “made me want to have a cigarette”).

Regarding the 2 images with movement, all participants thought the movement element (smoke) of the lit cigarette looked realistic and moved in a realistic way. All but 2 participants thought the color and density of the smoke were realistic. One participant thought the smoke was too dark at the top, while the other thought it was too light. For the soda bottle with condensation dripping and effervescence, 7 of the 10 participants thought the movement elements looked realistic, while 3 did not really notice the movement. Among the participants who noticed the condensation movement, 6 said the droplets were perfect in size and transparency.

Participants’ recommendations for additional smoking-related images to include in the future were largely secondary or distal cues, that is, they suggested items that often co-occur with smoking but that are not necessary for smoking itself (coffee, alcohol, a joint, food, phones, car, crowds, TV, and other smokers).

## Discussion

### Principal Results

Twelve smoking and nonsmoking augmented reality images were created using the Unity platform, Blender, and a combination of digital photography and extrusion modeling. Average participant ratings were all above 6 on a 10-point scale, suggesting that the images were reasonably realistic and coexisted in the overall environment well. Although this development and usability study was not powered for inferential comparisons, the urge to smoke following the visualization of smoking-related images was rated significantly higher than that following the visualization of non–smoking-related images, with a robust effect size. This finding is consistent with the existing literature on cue reactivity using in vivo items [30], pictures [21], and virtual reality cues [31].

In the open-ended usability interviews, the participants found the images to be generally realistic and detailed. However, there were numerous issues with image stability, lighting, shadows, and sizing. A number of participants reported that image details were either missing or different from their brand, which may be a consequence of our design choice for items to not look like any specific brand but rather be representative of the class of object (to be generalizable to a range of individuals). Based on participant feedback and experimenter observation, we developed a set of “best practice” recommendations to maximize the quality of the augmented reality experience (Table 3).

**Table 3:** Best practices for placement and viewing of augmented reality images.

Problem	Description	Solution(s)
Image placement	Difficulty for the app in locking an image into place on a surface	Use a surface with some variation or texture (eg, woodgrain) rather than glossy or uniformly white or black. When choosing where to place the image, try to have the edge of the surface (eg, table) in the screen. Do not have the smartphone camera lens close to and perpendicular to the plane onto which the image is to be placed. Do not place the object with the camera lens facing directly down (phone looking directly down on the object). If there are other objects on the table, do not attempt to place the augmented reality object too near the real object or the app may have difficulty identifying the correct plane and the augmented reality object may “jump on” the item.
Image stability	Shaking, moving, floating away	Do not have the smartphone camera lens close to and perpendicular to the plane onto which the image has been placed. Do not move the smartphone lens too close to the image (ie, so that it appears one is millimeters away from the object).
Light and shadow	Light reflection looking unnatural or static Lack of shadow or shadow that does not move naturally as the object is viewed from different angles.	Lighting conditions in the room in which the image was viewed appeared to affect the realism of the light and shadow on the object. Very bright rooms highlighted any deficiencies in shadowing of the object. Items that often reflect light (eg, metal pen) were more likely to have pixilation problems in the areas that were meant to reflect light.
Sizing	Image, once placed, is either much too large or small.	Scale appears to be affected by identifying the size of the plane through edges, intersecting walls, etc. Sizing problems were more common on large uniform surfaces where edges or walls were not in the screen view.

Many of the difficulties mentioned above may be resolved as augmented reality hardware and software advances, and some may already be addressable with more extensive programming and budgets to support it. Although other platforms, including Autodesk Maya and Autodesk 3ds Max, do provide additional capabilities, the developers used Blender, as it was the most suitable low-cost option.

### Limitations

There are several limitations to this development and usability study. First, although the augmented reality app can be used with any iPhone operating system device with augmented reality capabilities, a very recent model with an oversized screen was used in this study. Older models, smaller screens, or Android devices may have a different user experience in image quality or stability. Second, the smoking-related images chosen were a small sample of potentially relevant smoking stimuli. Although we selected items that are common to most smokers, there may be more potent triggers for some individuals, such as coffee and cigarette rolling papers. Likewise, the images that we chose were designed to be generic (ie, not tied to any specific brand, so as to be equally familiar to all participants). Brand-specific items may better elicit urges to smoke and appear more realistic to participants. Third, it is possible that the images intended to be neutral might nevertheless have elicited smoking urges in some participants. For example, if soda consumption had been reliably paired with smoking, the soda bottle could have been a conditioned stimulus rather than a neutral cue. Further, the laboratory setting in which participants viewed the augmented reality images was most likely dissimilar to their naturalistic smoking environments, which could have affected the ratings of smoking urges and reality/coexistence. Of course, the advantage of augmented reality is that the stimuli ultimately can be presented in smokers’ true smoking settings. Fourth, the results of this study may be limited by the small sample size of the participants.

### Conclusions

To our knowledge, this is the first application of augmented reality technology to create smoking stimuli that can be further evaluated for cue exposure therapy for smoking cessation. Smoking-related augmented reality images were successfully created and demonstrated to elicit urges to smoke, which were very similar to previous findings with in vivo cues [20-24]. Thus, it should be possible to use these images to extinguish smoking urges during quit attempts in locations associated with smoking in the real world. This has the potential to increase the long-term efficacy of cue exposure therapy. The next step toward the development of a functional smoking cessation augmented reality app is to test both cue reactivity and extinction of urge to smoke (craving) in a carefully controlled, fully powered experimental setting. This would be followed by continued testing in smokers’ naturalistic environments and by the addition of other user-friendly app features designed to improve ease of use and treatment adherence. The inclusion of an app readily accessible to smokers may improve the reach of cue exposure therapy. Ultimately, augmented reality technology may prove to facilitate cue exposure treatment and to be an effective, portable, adjuvant to treatments for dependence on tobacco as well as other substances.

## Appendix

Multimedia Appendix 1 Video of the augmented reality app in action.

Multimedia Appendix 2 Mean urge and reality/coexistence ratings of smoking-related augmented reality images.

Multimedia Appendix 3 Mean urge and reality/coexistence ratings of neutral augmented reality images.

## Abbreviations

CO: carbon monoxide
FTND: Fagerström Test for Nicotine Dependence
