# Supplementary figures and images for: Augmented Reality for Smoking Cessation: Development and Usability Study
Source: JMIR Mhealth Uhealth. 2020 Dec 31;8(12):e21643. doi: 10.2196/21643 (PMC7808889; doi:10.2196/21643)

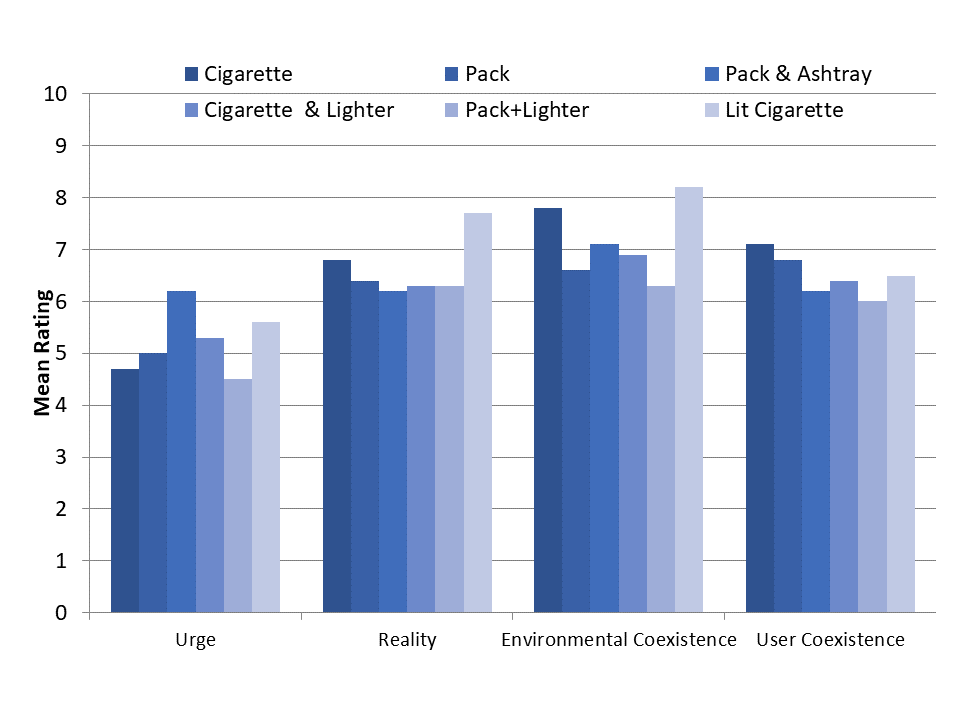

Supplement: Multimedia Appendix 2 [file mhealth_v8i12e21643_app2.png]

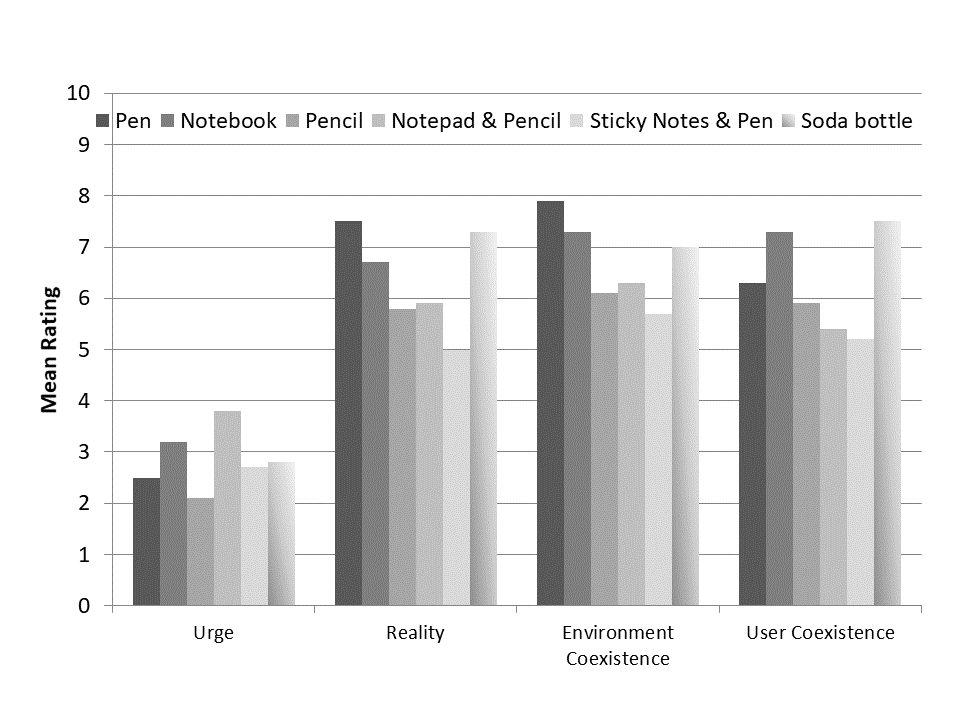

Supplement: Multimedia Appendix 3 [file mhealth_v8i12e21643_app3.png]
